# Functional form estimation using oblique projection matrices for LS-SVM regression models

**DOI:** 10.1371/journal.pone.0217967

**Published:** 2019-06-07

**Authors:** Alexander Caicedo, Carolina Varon, Sabine Van Huffel, Johan A. K. Suykens

**Affiliations:** 1 Department of Applied Mathematics and Computer Science, Faculty of Natural Sciences and Mathematics, Universidad del Rosario, Bogota, Colombia; 2 Department of Electrical Engineering ESAT-STADIUS Center for Dynamical Systems, Signal Processing, and Data Analytics/KU Leuven, Belgium; 3 imec, Leuven, Belgium; Institut de Robotica i Informatica Industrial, SPAIN

## Abstract

Kernel regression models have been used as non-parametric methods for fitting experimental data. However, due to their non-parametric nature, they belong to the so-called “black box” models, indicating that the relation between the input variables and the output, depending on the kernel selection, is unknown. In this paper we propose a new methodology to retrieve the relation between each input regressor variable and the output in a least squares support vector machine (LS-SVM) regression model. The method is based on oblique subspace projectors (ObSP), which allows to decouple the influence of input regressors on the output by including the undesired variables in the null space of the projection matrix. Such functional relations are represented by the nonlinear transformation of the input regressors, and their subspaces are estimated using appropriate kernel evaluations. We exploit the properties of ObSP in order to decompose the output of the obtained regression model as a sum of the partial nonlinear contributions and interaction effects of the input variables, we called this methodology Nonlinear ObSP (NObSP). We compare the performance of the proposed algorithm with the component selection and smooth operator (COSSO) for smoothing spline ANOVA models. We use as benchmark 2 toy examples and a real life regression model using the concrete strength dataset from the UCI machine learning repository. We showed that NObSP is able to outperform COSSO, producing stable estimations of the functional relations between the input regressors and the output, without the use of prior-knowledge. This methodology can be used in order to understand the functional relations between the inputs and the output in a regression model, retrieving the physical interpretation of the regression models.

## Introduction

Non-parametric regression is an important field of data analysis. These non-parametric models use some observations of the input data and the desired target to estimate a function and make predictions [1, 2]. However, generally, these models focus on the prediction of the target variable of interest and not on the model interpretability. In this manuscript, we will refer to interpretability as the property of a model to express the output in additive terms of the partial nonlinear contributions of the input variables and their interaction effects. In several applications interpretability plays an important role in the construction of prediction models. In such cases, the main goal is not the prediction of the response of a system but to determine the underlying mechanisms and the relationship between the inputs and the output. For instance, in medical applications, this information can be used in order to identify treatment targets, support diagnosis, and facilitate the introduction of these models in clinical practice. As an example, Van Belle et al. proposed the use of a color code to enhance the interpretability of classification models for the clinicians [3].

Interpretability of black box models has already been addressed for classifiers and regression models using different strategies. On the one hand, Van Belle et al. proposed a clinical interpretable classification model based on a least squares support vector machine (LS-SVM) using the radial basis function (RBF) kernel [4]. In that work, the authors retrieved the interpretability of the classifiers by using a truncated multinomial expansion of the RBF kernel. On the other hand, for regression models, interpretability has been tackled by using sparse additive models [5], and functional ANOVA models [6, 7]. In these models the target observations are modeled as a sum of a constant term, main effects, and interaction effects. In this context, the main effects refer to the direct relation between each input variable and the output, considering both the linear and nonlinear contributions of an input regressor on the output, while the interaction effect refers to the combined effect of 2 or more input variables on the output [6]. These models retrieve interpretability similarly to the case of generalized linear models [8], where a direct link between the contribution of each input in the output is estimated explicitly. However, these models require the use of prior knowledge in terms of which variables should be included in the regression models, as well as which interaction effects are of importance. This can only be achieved if the designer has a profound knowledge of the process and mechanisms underlying the changes in the target variable. To address this problem, some methodologies have been developed in order to select components that are relevant for the regression model in an automatic way, [9–11]. In particular, in the work from Lin et al., a new method for model selection and fitting in non-parametric regression models using smoothing spline ANOVA is proposed [11]. This method is referred as the Component Selection and Smoothing Operator (COSSO). This methodology is able to produce a model that is accurate in the prediction of the target variable, and it retrieves some of the interpretability by accurately identifying the components and functional forms of the relation between the input variables and the output. Nevertheless, it requires to specify a-priori if the user is interested in finding only main effects or interaction effects as well. More importantly, the solution of both problems does not converge, since the common terms in both cases are not the same. This is due to the fact that both problems, computing the main effect or including the interaction effects, lead to different cost functions with the same objective, fit the observed output. Additionally, this method has been developed for smoothing spline ANOVA models (SS-ANOVA) and, to the best of our knowledge, it has not been extended to other kernel based regression models.

From a geometry point of view, interpretability of non-parametric regression models can be addressed as the decomposition of a target observation vector into additive components [12]. Each of these components should lie in the subspaces that are spanned by the respective input regressors. If a basis for the subspaces of each individual input regressor and their interaction effects can be found, then appropriate projection matrices can be constructed in order to retrieve the interpretability of the models. This idea has been previously exploited in the case of linear models, where we have proposed to use a Hankel expansion of the input variables to construct a basis for their subspaces, and construct oblique subspace projectors in order to effectively decouple the dynamics of undesired input variables, representing the output as a sum of the partial linear contributions of each input [13]. Oblique projectors are particularly powerful when the variables that want to be decoupled are not orthogonal [14]. They have been applied as a preprocessing step in different hyper-spectral imaging [15], decoding [16], and biomedical applications [13].

Considering non-linear regression models using LS-SVM, due to the geometry of the RBF kernel, the subspaces spanned by the nonlinear transformation of the input regressors are not likely to be orthogonal [17]. Therefore, in order to decompose the output into interpretable additive components, oblique projectors should be used. In this paper we address this issue, by formulating a nonlinear extension to oblique subspace projections. We show how to create a basis for the subspaces spanned by the nonlinear transformation of the input regressors, main effects as well as interaction effects, using kernel evaluations. In addition, we suggest how to construct appropriate oblique projectors in order to effectively decompose the output of a kernel based regression model into interpretable additive components.

The manuscript is structured as follows, in section 1 we briefly describe an LS-SVM regression model. In section 2 we describe how to construct oblique subspace projectors and we present the proposed algorithm. In section 3 we evaluate the performance of the proposed algorithm using 2 toy examples and an application using the concrete strength dataset from the UCI machine learning repository. In section 4 we discuss the results and the potential use of the proposed algorithm, and we finalize with some concluding remarks in section 5.

Throughout this manuscript we will use the following notation: we will refer to scalars as italic letters (*x*), vectors will be represented as lowercase bold variables (**x**), matrices will refer to capital bold variables (**Ω**), subspaces will be represented by calligraphic capital letters (V), and spaces will be represented by blackboard bold capital letters (R). With some abuse of notation, we will refer to the projector matrix onto the subspace of the *l*^th^ input regressors, along the subspace spanned by the other regressors as **P**_*l*/(*l*)_, where the subindex *l* represents the subspace of the regressor where the output will be projected, the subindex (*l*) represents all the input regressors excluding the *l*^th^, and the symbol / represents the oblique projection.

## 1 LS-SVM for nonlinear regression

LS-SVM is a kernel based methodology that can be used to solve nonlinear classification and regression problems [18]. Due to its flexibility to manage different kind of problems and produce an adequate mathematical model, LS-SVM has been used successfully in different application fields such as: the prediction of electricity energy consumption [19], estimation of water pollution [20], forecasting of carbon price [21], and the prediction of meteorological time series [22], among others. However, its applications have been hampered due to its black-box model nature.

Let’s consider the following nonlinear regression model:

$$\begin{array}{c} \hat{y}\left(x\right)=w^{T}\varphi \left(x\right)+b, \end{array}$$ (1)

where $\hat{y}\in R$ represents the output of the model, $x\in R^{d}$, and **x** = [*x*^(1)^; …; *x*^(*d*)^], represents the input vector, *x*^(*l*)^ is the entry for the *l*^th^ input regressor, $\varphi \left(\cdot \right):R^{d}\to R^{p}$ represents the nonlinear mapping of **x** into a possibly infinite-dimensional feature space, ***w*** are the weigths, and *b* is the bias term. Given the following training data ${\left\{x_{i},y_{i}\right\}}_{i=1}^{N}$, the LS-SVM regression problem can be formulated as follows:

$$\begin{array}{c} \begin{array}{cc} \min_{w,e,b}J\left(w,e\right)= & \frac{1}{2}w^{T}w+\gamma \frac{1}{2}\sum_{i=1}^{N}e_{i}^{2}\\ \text{s.t.} & y_{i}=w^{T}\varphi \left(x_{i}\right)+b+e_{i},\;i=1,\ldots ,N, \end{array} \end{array}$$ (2)

where *γ* represents the regularization constant, ***e*** = [*e*_1_; …; *e*_*N*_] is the error vector with *e*_*i*_ the error related to the *i*^th^ observation. By taking the Lagrangian and solving for the Karush-Kuhn-Tucker conditions for optimality, the solution is given by:

$$\left[\begin{array}{cc} 0 & {1_{\text{N}}}^{T}\\ 1_{\text{N}} & \Omega +\frac{1}{\gamma }\text{I} \end{array}\right]\left[\frac{b}{\alpha }\right]=\left[\frac{0}{\text{y}}\right]$$ (3)

where **y** = [*y*_1_; …; *y*_*N*_], **1**_**N**_ = [1; …; 1], **Ω**_*ij*_ = *φ*(**x**_*i*_)^*T*^ *φ*(**x**_*j*_) = *K*(**x**_*i*_, **x**_*j*_) is the *ij*–th entry of the kernel matrix **Ω**, *K*(⋅, ⋅) is the kernel function, and ***α*** = [*α*_1_; …; *α*_*N*_] are the Lagrange multipliers, and *b* is the bias term.

The matrix form of the solution, for the training points, is then given by:

$$\begin{array}{c} \begin{array}{c} \hat{y}=\Omega \alpha +b, \end{array} \end{array}$$ (4)

with $\hat{y}=\left[{\hat{y}}_{1};\ldots ;{\hat{y}}_{N}\right]$, and **Ω**_*ij*_ = K(**x**_*i*_, **x**_*j*_).

Since the algorithm that is proposed in this paper makes use of projection matrices, it is important that the data that is projected is centered. This means, that the bias term in the regression model should be eliminated, as well as the mean value of the nonlinear transformation of the input regressors should be removed. These modifications lead to the following solution in matrix form [23]:

$$\begin{array}{c} \begin{array}{c} \hat{y}=\Omega_{C}\alpha . \end{array} \end{array}$$ (5)

where **Ω**_*C*_ = **MΩM**, is the centered kernel matrix, and $M=I-1_{N}1_{N}^{T}/N$ is a centering matrix. The *ij*–th entry of the centered kernel matrix is given by $\Omega_{C_{ij}}=(\varphi \left(x_{i}\right)-\mu_{\varphi }{)}^{T}(\varphi \left(x_{j}\right)-\mu_{\varphi })$.

## 2 Nonlinear regression decomposition using ObSP

In this section we present the propossed decomposition algorithm, which is a nonlinear extention to oblique subspace projections (NObSP). NObSP is not a regression method but an algorithm that allows to decompose the output of a regression model, using LS-SVM regression, into additive components that represent the partial nonlinear contributions of the input regressors on the output, and their interaction effects. NObSP uses oblique subspace projections to extract the partial contribution of an input regressor, while nullifying the contributions from other regressors. We will first introduce the concept of oblique subspace projections, then we will proposed their extention for nonlinear regression models.

### 2.1 Oblique subspace projections

Oblique subspace projection (ObSP) is a generalization of orthogonal projectors, where a given vector is projected onto a target subspace following the direction of a reference subspace [24]. Oblique subspace projectors can be used in order to decompose a signal into the partial contributions of some regressors, even when the signal subspaces of the different input regressors are not orthogonal [13, 16]. An algorithm for the use of ObSP in signal decomposition for linear regression models has been proposed in [13]. Briefly, the algorithm is summarized as follows. Let’s define $V\subset R^{N}$ as the subspace spanned by a matrix **A** = [**A**_*l*_ **A**_(*l*)_], with $A\in R^{N\times p}$, $A_{l}\in R^{N\times q}$ the partition of **A** that spans the subspace $V_{l}\subset V$, and $A_{\left(l\right)}\in R^{N\times (p-q)}$ the partition of **A** that spans the subspace $V_{\left(l\right)}\subset V$, $V_{\left(l\right)}\equiv \text{Span}\left(A_{\left(l\right)}\right)$, such that $V=V_{l}⊕V_{\left(l\right)}$. Now, let’s consider $V=V_{1}⊕V_{2}⊕...⊕V_{d}$, with ⊕ being the direct sum operator, and *d* represents the number of signal subspaces embedded in **A** satisfying *d* ≤ *p*; then the oblique projector onto $V_{l}$ along $V_{\left(l\right)}=V_{1}⊕...⊕V_{l-1}⊕V_{l+1}...⊕V_{d}$, denoted by *P*_*l*/(*l*)_, is given by:

$$\begin{array}{c} P_{l/(l)}=A_{l}{\left(A_{l}^{T}Q_{\left(l\right)}A_{l}\right)}^{†}A_{l}^{T}Q_{\left(l\right)}, \end{array}$$ (6)

where † represents the generalized inverse, and **Q**_(*l*)_ is the orthogonal projector onto Null $\left(A_{\left(l\right)}^{T}\right)\subset V_{\left(l\right)}^{⊥}$, which is computed as:

$$\begin{array}{c} Q_{\left(l\right)}=I_{N}-P_{\left(l\right)}, \end{array}$$ (7)

where $P_{\left(l\right)}=A_{\left(l\right)}{\left(A_{\left(l\right)}^{T}A_{\left(l\right)}\right)}^{†}A_{\left(l\right)}^{T}$ is the orthogonal projector onto $V_{\left(l\right)}$ [24].

### 2.2 Nonlinear oblique subspace projection (NObSP)

Let’s consider the following nonlinear regression problem:

$$\begin{array}{c} y\left(x\right)=\sum_{l=1}^{d}f_{l}\left(x^{\left(l\right)}\right)+\sum_{l=1}^{d}\sum_{h>l}^{d}f_{lh}\left(x^{\left(l\right)},x^{\left(h\right)}\right)+G\left(x\right)+e, \end{array}$$ (8)

where *e* is the error term, *f*_*l*_(*x*^(*l*)^) represents the partial contribution, linear and non-linear, of the *l*^th^ input variable *x*^(*l*)^ on the output, *f*_*lh*_(*x*^(*l*)^, *x*^(*h*)^) represents the partial contribution of the interaction between the variables *x*^(*l*)^ and *x*^(*h*)^ on the output, the term *G*(**x**) represents the partial contribution of all the other higher order interactions between the input variables, with **x** = [*x*^(1)^; …; *x*^(*d*)^], and *d* the number of input variables.

From Eq (8), it can be seen that the partial contributions of each input variable, their second order interactions, and the higher order interactions can be found if an appropriate projection matrix can be created. Such projection matrix will span the subspace of the nonlinear transformation of the (input signal)/(interaction effects) of interest, whilst its null subspace contains the nonlinear transformation of the other remaining input variables and their interactions. More especifically, if we define **P**_*i*/(*i*)_ as the oblique projection matrix onto the subspace spanned by the nonlinear transformation of the *i*^th^ input variable, along the direction defined by the other variables, then by multiplying Eq (8) by this projection matrix, we obtain:

$$\begin{array}{c} P_{i/(i)}y\left(x\right)=P_{i/(i)}\sum_{l=1}^{d}f_{l}\left(x^{\left(l\right)}\right)+P_{i/(i)}\sum_{l=1}^{d}\sum_{h>l}^{d}f_{lh}\left(x^{\left(l\right)},x^{\left(h\right)}\right)+P_{i/(i)}G\left(x\right)+P_{i/(i)}e. \end{array}$$ (9)

Since the oblique projection matrix nullifies all the nonlinear contributions of the input variables, except the *i*^th^ input variable, then Eq (9) reduces to:

$$\begin{array}{c} P_{i/(i)}y\left(x\right)=y^{\left(i\right)}\left(x\right)=f_{i}\left(x^{\left(i\right)}\right)+e^{\left(i\right)}, \end{array}$$ (10)

where *f*_*i*_(*x*^(*i*)^) is the partial contribution of the *i*^th^ input variable on the output, and *e*^(*i*)^ is an error term obtained by multiplying the original error by the projection matrix.

In the case of functional ANOVA models, it is assumed that these subspaces are orthogonal [6, 11]. This leads to orthogonal projection matrices. However, there is no evidence to support this claim, especially when using kernel regression models. In such cases, an oblique projector will be more appropriate. The problem now lies in finding a basis for the signals subspaces that are required to construct the oblique projector operator.

In a kernel regression framework, the output of the regression model can be expressed as a linear combination of kernel evaluations, i.e. $\hat{y}\left(x\right)=\sum_{i=1}^{N}\alpha_{i}K\left(x,x_{i}\right)+b$. Therefore, the kernel matrix spans the column space of the nonlinear transformation of the input variables. In order to see this, lets consider the nonlinear regression problem for the centered data in matrix form, **y** = **Φ**_*C*_ ***ω*** + ***e***, where **Φ**_*C*_ = [*φ*^*T*^(**x**_1_) − *μ*_*φ*_; …; *φ*^*T*^(**x**_*N*_) − *μ*_*φ*_], to solve that problem using least squares, the solution leads to the hat, or projection, matrix **P**_**C**_ = **Φ**_***C***_(**Φ**_***C***_^*T*^ **Φ**_***C***_)^−1^ **Φ**_***C***_^*T*^, thereby $\hat{y}=\text{Py}$. The projection matrix can also be written using the centered kernel matrix **Ω**_*C*_, in this case the projection matrix has the form **P**_**C**_ = **Ω**_***C***_(**Ω**_***C***_^*T*^ **Ω**_***C***_)^−1^ **Ω**_***C***_^*T*^, To prove this we can replace in the previous equation $\Omega_{C}=\Phi_{C}\Phi_{C}^{T}$, which after some algebraic manipulation leads to **P**_**C**_ = **Φ**_***C***_(**Φ**_***C***_^*T*^ **Φ**_***C***_)^−1^ **Φ**_***C***_^*T*^. Taking this into account, if we consider the matrix **Φ**_***C****l*_ representing the centered nonlinear transformation of the *l*^th^ input regressor and defined as ${\Phi_{C}}_{l}=\left[\varphi {\left(0,\ldots ,x_{1}^{\left(l\right)},\ldots ,0\right)}^{T}-\mu_{\varphi };\ldots ;\varphi {\left(0;\ldots ,x_{N}^{\left(l\right)},\ldots ,0\right)}^{T}-\mu_{\varphi }\right]$, then, the projection matrix onto its subspace is given by **P**_**C***l*_ = **Φ**_***C****l*_(**Φ**_***C****l*_^*T*^**Φ**_***C****l*_)^−1^**Φ**_***C****l*_^*T*^, since the nonlinear transformation is not known, we cannot directly compute **P**_**C***l*_ but we propose to use kernel evaluations as follows:

**Proposition 1**. *Let* **y** = **Φ**_*C*_ ***w*** + **e**, *where* **Φ**_*C*_ = [*φ*(**x**_1_)^*T*^ − *μ*_*φ*_; …; *φ*(**x**_*N*_)^*T*^ − *μ*_*φ*_] *is a matrix containing the centered nonlinear transformation of the regressor variables* $x_{i}=\left[x_{i}^{\left(1\right)};\ldots ;x_{i}^{\left(d\right)}\right]$. *Using LS-SVM the solution to this problem is given by* $\hat{y}=\Omega_{C}\alpha$, *with* **Ω**(*i*, *j*) = *K*(**x**_*i*_, **x**_*j*_), *K*(⋅, ⋅) *the kernel function*, **Ω**_*C*_ = **MΩM**, *and* $M=I-1_{N}1_{N}^{T}/N$. *Then, the kernel matrix* **Ω**_**C***l*_, *formed using K*(**x**^(*l*)^, **x**), *with* **x**^(*l*)^ = [0; …; *x*^(*l*)^; …; 0] *a vector containing only the l^th^ element of the vector* **x** *for l* ∈ {1, …, *d*}, *spans the subspace of the centered nonlinear transformation of the l^th^ input regressor*.

*Proof*. Consider ${\Phi_{C}}_{l}=\left[\varphi {\left(0;\ldots ;x_{1}^{\left(l\right)};\ldots ;0\right)}^{T}-\mu_{\varphi };\ldots ;\varphi {\left(0;\ldots ;x_{N}^{\left(l\right)};\ldots ;0\right)}^{T}-\mu_{\varphi }\right]$ with ${{\text{P}}_{\text{C}}}_{l}={\Phi_{C}}_{l}{\left({{\Phi_{C}}_{l}}^{T}{\Phi_{C}}_{l}\right)}^{-1}{{\Phi_{C}}_{l}}^{T}$ the projection matrix onto the subspace defined by the centered nonlinear transformation of the *l*^th^ regressor variable. Defining ${\Omega_{C}}_{l}={\Phi_{C}}_{l}{\Phi_{C}}^{T}$, and using ${{\text{P}}_{\text{C}}}_{l}={\Omega_{C}}_{l}{\left({\Omega_{C}}_{l}^{T}{\Omega_{C}}_{l}\right)}^{-1}{\Omega_{C}}_{l}^{T}$, this leads to ${{\text{P}}_{\text{C}}}_{l}={\Phi_{C}}_{l}{\Phi_{C}}^{T}{\left(\Phi_{C}{\Phi_{C}}_{l}^{T}{\Phi_{C}}_{l}{\Phi_{C}}^{T}\right)}^{-1}\Phi_{C}{\Phi_{C}}_{l}^{T}$. Using the SVD decomposition of the matrix **Φ**_***C***_, **Φ**_***C***_ = **UΛV**^*T*^, and replacing in the definition of ${{\text{P}}_{\text{C}}}_{l}$ we obtain ${{\text{P}}_{\text{C}}}_{l}={\Phi_{C}}_{l}{\left(U\Lambda V^{T}\right)}^{T}{\left(U\Lambda V^{T}{\Phi_{C}}_{l}^{T}{\Phi_{C}}_{l}{\left(U\Lambda V^{T}\right)}^{T}\right)}^{-1}\left(U\Lambda V^{T}\right){\Phi_{C}}_{l}$, which after some algebraic manipulations leads to ${{\text{P}}_{\text{C}}}_{l}={\Phi_{C}}_{l}\left(V\Lambda U^{T}\right){\left(V\Lambda U^{T}\right)}^{-1}{\left({\Phi_{C}}_{l}^{T}{\Phi_{C}}_{l}\right)}^{-1}{\left(U\Lambda V^{T}\right)}^{-1}\left(U\Lambda V^{T}\right){\Phi_{C}}_{l}$, and reducing we finally obtain ${{\text{P}}_{\text{C}}}_{l}={\Phi_{C}}_{l}{\left({{\Phi_{C}}_{l}}^{T}{\Phi_{C}}_{l}\right)}^{-1}{{\Phi_{C}}_{l}}^{T}$.

In the same way we can use *K*(**x**^([*l*])^, **x**), with **x**^([*l*])^ = [*x*^(1)^; …; *x*^(*l*−1)^; 0; *x*^(*l*+1)^; …; *x*^(*d*)^] to form the kernel matrix **Ω**_*C*__(*l*)_ which can be used as a basis for the subspace spanned by the centered nonlinear transformation of all the other input regressor variables, excluding the *l*–th input regressor, as well as their interaction effects. It is important to notice that the subspace spanned by the nonlinear transformation of more than one input regressor will contain their interaction effects as well as their individual contribution, or main effect. Hence, if the interest is to find solely the interaction effect between 2 variables on the output, their individual contributions should be found first and subtracted. For instance, if we define **P**_*ij*/(*ij*)_ as the oblique projection matrix onto the subspace spanned by the nonlinear transformations of the *i*^th^ and *j*^th^ input variables, along the direction defined by the other variables, then by multiplying Eq (8) by this projection matrix, we obtain:

$$\begin{array}{c} P_{ij/(ij)}y\left(x\right)=P_{ij/(ij)}\sum_{l=1}^{d}f_{l}\left(x^{\left(l\right)}\right)+P_{ij/(ij)}\sum_{l=1}^{d}\sum_{h>l}^{d}f_{lh}\left(x^{\left(l\right)},x^{\left(h\right)}\right)+P_{ij/(ij)}G\left(x\right)+P_{ij/(ij)}e. \end{array}$$ (11)

which reduces to:

$$\begin{array}{c} P_{ij/(ij)}y\left(x\right)=y^{\left(ij\right)}\left(x\right)=f_{i}\left(x^{\left(i\right)}\right)+f_{j}\left(x^{\left(j\right)}\right)++f_{ij}\left(x^{\left(ij\right)}\right)+e^{\left(ij\right)}, \end{array}$$ (12)

where *f*_*i*_(*x*^(*i*)^) and *f*_*j*_(*x*^(*j*)^) are the partial contribution of the *i*^th^ and *j*^th^ input variables on the output, *f*_*ij*_(*x*^(*ij*)^) is the nonlinear contribution of the interacion effect of the *i*^th^ and *j*^th^ input variable, and *e*^(*ij*)^ is an error term obtained by multiplying the original error by the projection matrix. Therefore, in order to obtain only the interaction effect, the individual partial contributions should be subtracted.

Once the basis for the main contributions, and interaction effects, of given input regressors have been found, the output of an LS-SVM regression model can be decomposed into additive components using Algorithm I.

### 2.3 Out-of-sample extension

In **Algorithm I** we presented a way to derive the main and interaction effect contributions using training data. This idea can be extended to data that has not been seen during training, however some considerations need to be taken into account. Since the algorithm that is presented in this paper is based on projection matrices, it is important to notice that a proper set of basis vectors are needed in order to construct reliable projection matrices. For instance, if while training the algorithm, it is found that the dimensions of the subspace where the data lies is *N*_*d*_, then at least *N*_*d*_ basis vectors are needed to construct a proper projection matrix. In addition, the number of data points used to evaluate the model will define the size of the kernel matrix, which defines the maximum number of basis vectors that can be obtained from it. Therefore, in order to construct projection matrices for data-points outside the training set, and considering *N*_*d*_ as the dimension of the subspace of interest, then at least *N*_*d*_ new data points are needed in order to properly decompose the output into the nonlinear partial contributions. In practice, while decomposing the training data, the dimension of each one of the subspaces that represent the nonlinear transformation of the input variables, as well as the interaction effects, should be computed. The maximum dimension of such subspaces can be taken as the minimum number of evaluation points that are needed in order to produce a proper decomposition. The steps needed to evaluate NObSP using new data points are summarized in the Algorithm II.

**Algorithm I**. **Nonlinear oblique subspace projections (NObSP)**

**Input**: regressor matrix $X\in R^{N\times d}$, output vector $y\in R^{N}$, estimated output vector $\hat{y}\in R^{N}$.

**Output**: ${\hat{y}}^{\left(l\right)}\in R^{N}$, and $y^{\left(l\right)}\in R^{N}$ main effect and interaction effect contributions from given input regressors using the estimated output from the model and the real measured output, respectively.

1. Normalize the input regressors.

2. Train an LS-SVM regression model using the training input/output data {**X**, **y**}.

3. Compute the kernel matrices **Ω**_*l*_ and **Ω**_(*l*)_, representing the subspaces spanned by the regressor(s) of interest, as explained in **Proposition 1**.

4. Center the Kernel matrices as follows: **Ω**_*C*_ = **MΩM**, where **M** = **I** − **1**_*N*_ **1**_*N*_^*T*^ / *N* is a centering matrix, with **I** the identity matrix, **1**_*N*_ a column vector of ones, and *N* the number of training data points.

5. Compute the oblique projector as in Eq (6) using the centered **Ω**_*l*_ and **Ω**_(*l*)_, $P_{l/(l)}=\Omega_{l}{\left(\Omega_{l}^{T}Q_{\left(l\right)}\Omega_{l}\right)}^{†}\Omega_{l}^{T}Q_{\left(l\right)}$, with $Q_{\left(l\right)}=I_{N}-\Omega_{\left(l\right)}{\left(\Omega_{\left(l\right)}^{T}\Omega_{\left(l\right)}\right)}^{†}\Omega_{\left(l\right)}^{T}$.

6. Compute the corresponding partial contribution to the output as ${\hat{y}}^{\left(l\right)}=P_{l/(l)}\hat{y}$, or **y**^(*l*)^ = **P**_*l*/(*l*)_**y** with *l* = 1, …, *d*.

The output from both algorithms can be used in order to determine which input regressor(s) and/or interaction effects are of importance in the output of the model, by computing the percentage of power that each one of these contributions provide to the output.

## 3 Applications

In this section we present results from the proposed algorithm using 2 simulation examples, as well as a real life example using data from the Concrete Compressive Strength Data Set in the UCI machine learning repository [25]. In the first toy example we compare NObSP with the results given by COSSO, using the same example proposed in [11], where only main effects are included. Additionally, we evaluate the performance of NObSP to select relevant components, and we test the effect of the kernel selection in the decomposition. In the second example, we create an artificial dataset that includes interaction effects, we test for the robustness of the projections and the regression model by means of bootstrapping, we present the results in terms of their mean solution and 95% confidence intervals, in this example we also evaluated the performance of the model to unseen test data. In the third example we demonstrate the potential use of NObSP in a real life example.

**Algorithm II**. **Out-of-Sample Extension for NObSP**

**Input**: regressor matrix with training samples $X_{train}\in R^{N_{train}\times d}$, output vector for training data $y_{train}\in R^{N_{train}}$, regressor matrix with test samples $X_{test}\in R^{N_{test}\times d}$, with *N*_*test*_ ⩾ *N*_*d*_, with *N*_*d*_ the largest dimension of the subspaces representing the nonlinear transformation of the input data.

**Output**: partial contribution and interaction effects from given input regressors in the test set ${\hat{y}}_{test}^{\left(l\right)}\in R^{N_{test}}$.

1. Normalize the input regressors, **X**_*train*_ and **X**_*test*_.

2. Train an LS-SVM regression model using the training input/output data {**X**_*train*_, **y**_*train*_}.

3. Compute the kernel matrices for the test set $\Omega_{l}^{\left(test\right)}$ and $\Omega_{\left(l\right)}^{\left(test\right)}$, by evaluating the kernel function using samples from the test set, with $\Omega_{l}^{\left(test\right)}\left(i,j\right)=K(x_{test}^{\left(l\right)}\left(i\right),x_{train}\left(j\right))$ the *ij*–*th* element of the kernel matrix, where $x_{test}^{\left(l\right)}\left(i\right)\in R^{1\times d}$ is the *i*–th row of **X**_*test*_ where all elements are zero except the *l*–th component, and $x_{train}\left(j\right)\in R^{1\times d}$ is the *j*–th row of **X**_*train*_. These matrices will represent the subspaces spanned by the regressor(s) of interest, as explained in **Proposition 1**.

4. Evaluate the output of the model, ${\hat{y}}_{\left(test\right)}$ using Eq (5).

5. Center the Kernel matrices as follows: $\Omega_{C}^{\left(test\right)}=\Omega^{\left(test\right)}-M_{1}\Omega^{\left(test\right)}-\Omega^{\left(test\right)}M_{2}+M_{1}\Omega^{\left(test\right)}M_{2}$, where $1_{N_{test}}$ is a column vector of ones, $M_{1}=1_{N_{test}}1_{N_{test}}^{T}/N_{test}\in R^{N_{test}\times N_{test}}$ is a centering matrix, $M_{2}=1_{N_{train}}1_{N_{train}}^{T}/N_{train}\in R^{N_{train}\times N_{train}}$ is a centering matrix, *N*_*train*_ is the number of training samples used, and *N*_*test*_ is the number of test samples used.

6. Compute the oblique projector as in Algorithm I, using the centered $\Omega_{l}^{\left(test\right)}$ and $\Omega_{\left(l\right)}^{\left(test\right)}$.

7. Compute the corresponding partial contribution in the output as ${\hat{y}}_{test}^{\left(l\right)}=P_{l/(l)}{\hat{y}}_{\left(test\right)}$, with *l* = 1, …, *d*.

### 3.1 Simulation study: Toy example I

In order to compare the performance of NObSP with COSSO, we use the first example presented in [11], where an additive model in $R^{10}$ is considered. The regression function is defined by *f*(**x**) = 5*g*_1_(*x*^(1)^) + 3*g*_2_(*x*^(2)^) + 4*g*_3_(*x*^(3)^) + 6*g*_4_(*x*^(4)^), where: *g*_1_(*x*) = *x*, *g*_2_(*x*) = (2 *x* − 1)^2^, $g_{3}\left(x\right)=\frac{\sin(2\pi x)}{2-2\sin(2\pi x)}$, and *g*_4_(*x*) = 0.1 sin(2*πx*) + 0.2 cos(2*πx*) + 0.3 sin^2^(2*πx*) + 0.4 cos^3^(2*πx*) + 0.5 sin^3^(2*πx*). The model contains 4 main effects, no interaction effects, and no influence from the inputs *x*^(5)^ until *x*^(10)^. We generated the input data, **x**, as uniformly distributed random numbers in [0, 1]. Additionally, we added noise such that the signal-to-noise ratio is 3: 1 as in [11]. In contrast with the original example, we also imposed a correlation between the input variables of 0.8 in order to increase the complexity of the problem. To impose this correlation we used another uniformly distributed variable *u*, and the following formula $x_{n}^{\left(i\right)}=\frac{x^{\left(i\right)}+tu}{1+t}$, where $t=\sqrt{\frac{\rho }{1-\rho }}$ and *ρ* is the desired correlation value that will be impossed on the variables. Since we are using uniformly distributed data, due to the central limite theorem, the resulting input variables will be closer to a normal distribution and will not be uniformly distributed. We use COSSO in 2 ways, first to find only the main effects in the regression, i.e. decompose the output only in additive terms of the partial nonlinear contributions of the input regressors, we will refer to this as COSSO-I. Second, we also computed the output using COSSO for the second order interactions, retrieving not only the main effects but also the interaction effect, to compare if both approaches for COSSO converge to the same result. We refer to this as COSSO-II.

**Fig 1:**
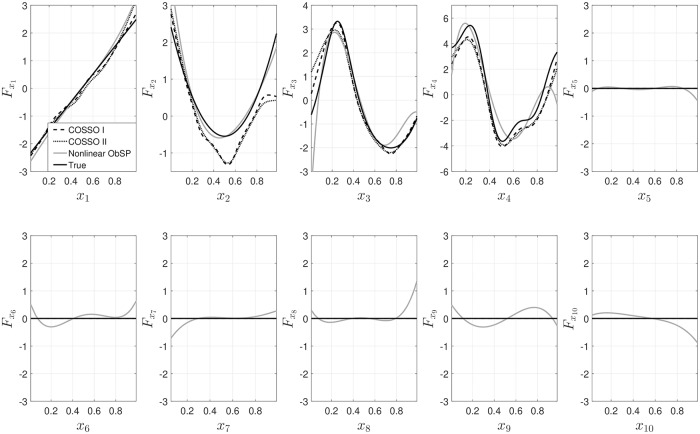
Results from the decomposition of the output for the toy example I using NObSP (gray solid line), COSSO I dashed black line, and COSSO II in a black dotted line. The solid black line represents the true component.

The results for the decomposition are shown in Fig 1. The results from NObSP are presented in a solid gray line, COSSO-I in a black dashed line and COSSO II in a black dotted line. The true output is shown as a black solid line. As can be seen in the Fig, the proposed algorithm is able to approximately retrieve the functional form for the contribution of each input regressor variable, with a performance similar to the one provided by COSSO. It is also possible to observe that the output provided by COSSO-I and COSSO-II are not the same. In addition, in contrast with COSSO, NObSP is not able to retrieve a response equal to zero for the contribution of the input variables that are not included in the final model. This is expected, since the proposed algorithm does not include any sparsity or feature selection criteria, instead it is solely based on the output of an existing regression model. However, the magnitude of the contribution of those regressors to the output is smaller than the contribution of the variables that are effectively included in the original model.

**Fig 2:**
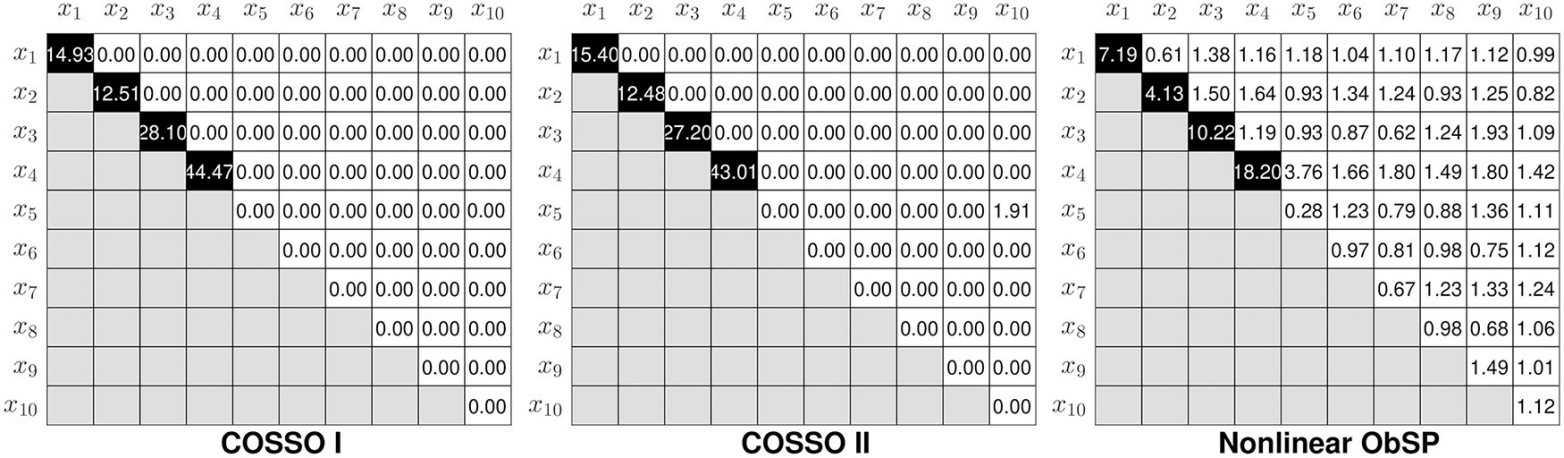
Percentage of the contributions of the input regressors, and their interaction effects, on the output. The components with a larger contribution in the final model are indicated by a black square.

In Fig 2, the strength of the nonlinear contributions of the input variables and the interaction effects are shown. To compute this strength, first we computed the total power of the decomposed signal as the sum of the root mean squared values from all the components obtained in the decomposition model. Once this is obtained, the strength of each component is computed as the ratio between its root mean squared value and the total power of the decomposed signal. To visualize the magnitude of the main nonlinear contributions and the interaction effects, we present these values in a matrix form. In this matrix the diagonal represents the strength of the contributions for each input variable, and the upper triangular elements represent the strength of the contribution for second order interactions for the corresponding input regressors, which are indicated in the rows and columns of the matrix. The lower triangular elements are not taken into account since they represent redundant information. Finally, the fields in the matrix that belong to the contributions with a larger influence on the output are colored in black.

From Fig 2 we can observed that COSSO-I only the diagonal produces elements different from 0, since it only retrieves main effects, while COSSO-II and NObSP produce a total of 55 components. It can be seen that the results from NObSP indicate that the first 4 components have a higher contribution to the output than the other components, with a contribution between [4.13%—18.20%] of the total power, compared to [12.48%—43.01%] produced by COSSO-II. COSSO-I also produces components with a larger magnitude in the first 4 components with a root mean square value of [12.51%—44.47%] and 0 in the other components. In contrast with NObSP, COSSO-I and COSSO-II produce components with a contribution equal to zero, due to its sparsity properties. Since NObSP does not include sparsity, most of its components contribute to the output. This results in lower strength values for the components with a higher influence in the output for NObSP. However, by selecting an appropriate threshold, these components can be selected. For this example the threshold was set to 4% by visual inspection of the components.

**Fig 3:**
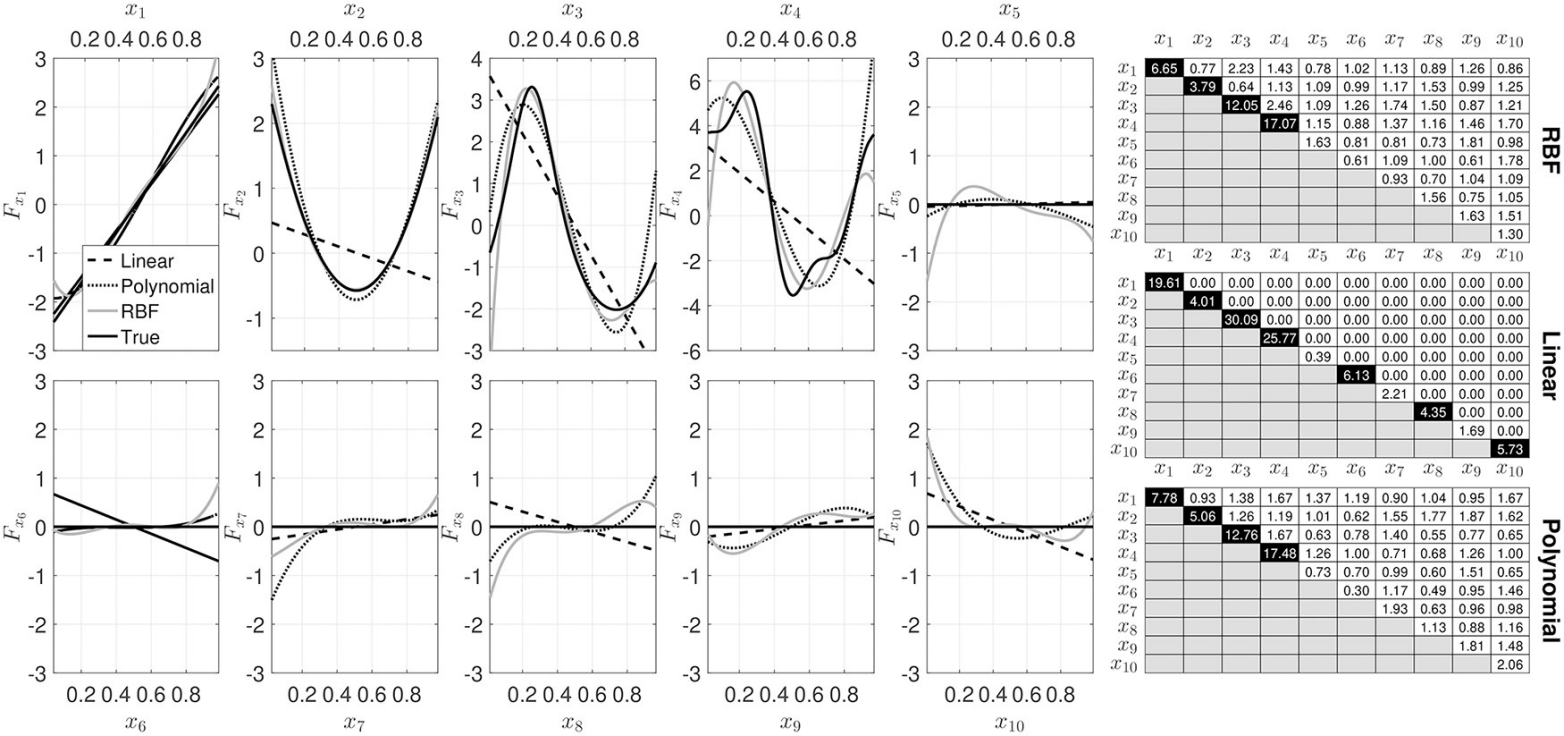
Output of NObSP using different kernels, in black dashed line for the linear kernel, in black dotted line for the polynomial kernel, and in gray solid line for the RBF kernel. The black solid line represents the true contribution.

In Fig 3 the results from the decomposition using NObSP with different kernels are shown. It can be seen that the linear kernel only produces the linear approximation of the nonlinear contributions of the input regressors on the output. Additionally, the contributions of the interaction effects, in the linear model, are equal to zero, which is expected since the interaction effects in linear models are given by a sum of the contribution of each input variable on the output. Therefore, by identifying such contributions and subtracting from the interaction effect the resulting component should be equal to zero. The performance of the polynomial kernel and the RBF kernel is quite similar, in both cases the decompositions were able to approximate the desired functional forms.

In Table 1 the root mean square errors for the regression, and the estimation of the functional forms using COSSO-I, COSSO-II and NObSP are shown. The simulations were performed 100 times and the values are presented as median [min-max]. It can be seen that COSSO-I and COSSO-II in general produced a lower error in the estimation of the overall function, with COSSO-I producing the lowest error. However, when estimating the functional forms of the main effects, NObSP, using the RBF kernel, outperforms the results from COSSO.

**Table 1:** Root mean square error for regression models and the estimation of the true functional forms using NObSP with three different kernels, COSSO-I, and COSSO-II. Values are expressed as median [min-max].

	*F*_**x**_	$F_{x_{1}}$	$F_{x_{2}}$	$F_{x_{3}}$	$F_{x_{4}}$	$F_{x_{5}}$	$F_{x_{6}}$	$F_{x_{7}}$	$F_{x_{8}}$	$F_{x_{9}}$	$F_{x_{10}}$
COSSO-I	**1.40[1.33-1.47]**	0.25[0.15-0.55]	0.53[0.41-0.78]	0.34[0.17-0.43]	1.30[1.08-1.40]	**0[0-0]**	**0[0-0.91]**	0[0-0.54]	**0[0-0.40]**	**0[0-0.20]**	**0[0-0]**
COSSO-II	1.55[1.39-1.59]	0.41[0.15-1.05]	0.54[0.51-0.63]	0.67[0.27-0.86]	1.49[1.26-1.57]	0[0-0]	0[0-1.02]	**0[0-0.52]**	0[0-0.71]	0[0-1.04]	**0[0-0]**
NObSP_Linear_	3.30[3.24-3.46]	0.39[0.16-0.47]	0.56[0.54-0.72]	0.97[0.91-1.00]	2.46[2.44-2.48]	0.31[0.15-0.50]	0.55[0.34-0.68]	0.20[0.04-0.25]	0.28[0.16-0.46]	0.02[0-0.19]	0.03[0-0.26]
NObSP_Poly_	2.78[2.65-2.78]	0.18[0.11-0.22]	0.24[0.16-0.35]	0.48[0.45-0.55]	1.27[1.21-1.29]	0.15[0.07-0.35]	0.25[0.16-0.37]	0.20[0.06-0.26]	0.20[0.08-0.31]	0.16[0.12-0.20]	0.26[0.11-0.48]
NObSP_RBF_	2.26[2.09-2.45]	**0.16[0.07-0.28]**	**0.19[0.12-0.27]**	**0.28[0.24-0.35]**	**0.86[0.84-0.88]**	0.18[0.13-0.38]	0.19[0.05-0.25]	0.14[0.09-0.34]	0.14[0.05-0.20]	0.17[0.10-0.31]	0.16[0.08-0.33]

### 3.2 Simulation study: Toy example II

For the second toy example, we use the following additive model in $R^{3}$:

$$\begin{array}{c} y=\sin\left(2\pi x_{1}\right)+1.4e^{x_{2}}+\cos(4\pi \left(x_{1}-x_{2}\right))+\eta ; \end{array}$$ (13)

with, *x*_1_, *x*_2_, *x*_3_ ∈ [0, 1], and *η* is a Gaussian noise with a variance such that the signal-to-noise ratio on the output variable is equal to 4db. No contribution from *x*_3_ was included. In addition, a correlation of 0.8 was imposed in the input signals.

We computed 400 realizations of the model. From this data, we extracted 1000 different training datasets using random sampling with replacement. For each generated dataset a model using LS-SVM was computed and tuned using 10-fold cross-validation. The partial contributions of the input variables and their second order interactions were calculated using NObSP and COSSO-II [11]. From the output of the 1000 randomizations, the mean and the 95% confidence intervals were computed. Additionally, we computed the output of the model for unseen data using **Algorithm II**. For this, we first found the rank for the different kernels obtained using **Algorithm I**, the maximum rank obtained was 45. Based on that rank we run simulations using a test set size from 1 data point up to 45 × 5 data points. For each test set size we generate 100 random sets. In order to identify the convergence of the error in the decomposition with respect to the test set size, we plotted the errors in terms of their mean and 95% confidence interval.

**Fig 4:**
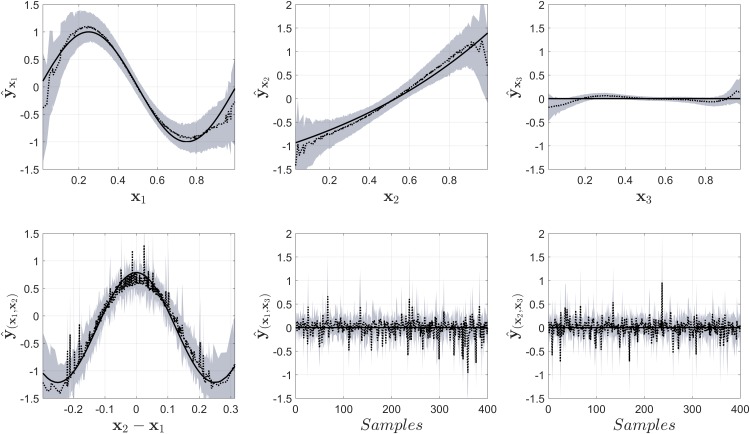
Output from NObSP, the black solid line represents the true component, the dotted black line represents the mean for the estimations using NObSP, and the gray area represents the 95% confidence interval obtaining using bootstrap.

In Fig 4 the results from NObSP using **Algorithm I** are shown. The gray area in the Fig represents the 95% confidence intervals, the dotted black line represents the mean value for the regression, while the black solid line represents the true component used as reference. This same convention will be used for the rest of the Fig. It is important to notice that the mean values plotted in the Figs do not represent the output of one decomposition of the regression models, but just the mean value of the output for that specific input value. This gives the impression that the models produce noisy estimates, but that is not the case. In the Fig it can be seen that NObSP is able to approximately retrieve the functional form of the simulated model. In addition, the contribution from the input *x*_3_ is small in amplitude compared to the other components, indicating that this variable does not contribute largely on the output. The second-order interactions appear to produce larger confidence intervals, indicated by the gray area. However, NObSP was still able to retrieve the approximate functional form of the second-order interaction between the variables *x*_1_ and *x*_2_, while producing a small output for the components that were not present in the original model.

**Fig 5:**
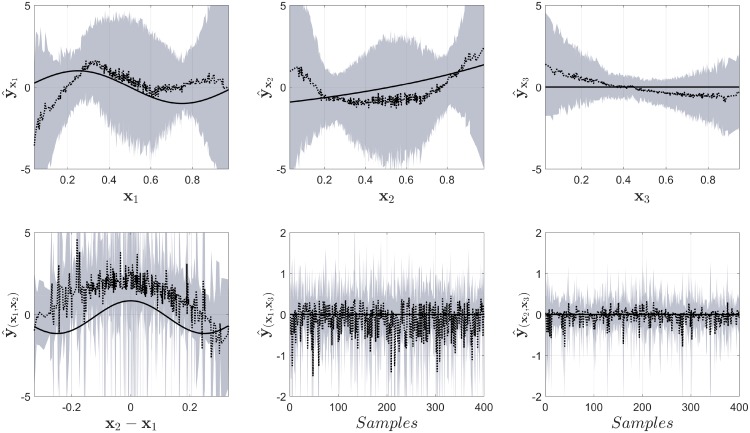
Output of the model for the decomposition of the measured output into the main and interaction effect contributions using COSSO-II in smoothing spline ANOVA models for second-order interactions. The black solid line represents the true component, the dotted black line represents the mean for the estimations using NObSP, and the gray area represents the 95% confidence interval obtained using bootstrap.

Fig 5 shows the results from the smoothing spline ANOVA models using COSSO-II. It can be seen that COSSO-II, in this case example, is not able to retrieve the desired functional forms. But, when looking at the error in the complete regression model the LS-SVM regression produce a root mean square error of 0.09 [0.02-0.11], while COSSO-II produce an error of 0.04 [0.03-0.05], indicating that COSSO II was able to fit better the model than NObSP. Here it is important to notice that NObSP is not a regression method, but just an algorithm that allows to decomposse the results from a regression model, using LS-SVM, into the partial nonlinear contributions of the input variables on the output, as well as their interaction effects. In contrast with NObSP, COSSO is embedded within the cost function of funtional ANOVA models. Therefore, COSSO migth lead to a better fit than the LS-SVM regression. However, this example shows that, enventhough the overal fit is worse, NObSP is able to retreive the funtional form of the nonlinear partial contributions of the input variables on the output, while COSSO fails.

**Fig 6:**
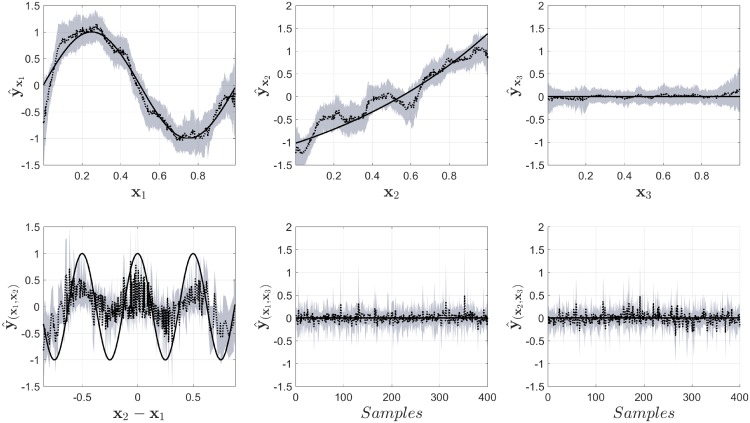
Output of the model for the decomposition using COSSO-II and non-correlated inputs. The black solid line represents the true component, the dotted black line represents the mean for the estimations using NObSP, and the gray area represents the 95% confidence interval obtaining using bootstrap.

**Fig 7:**
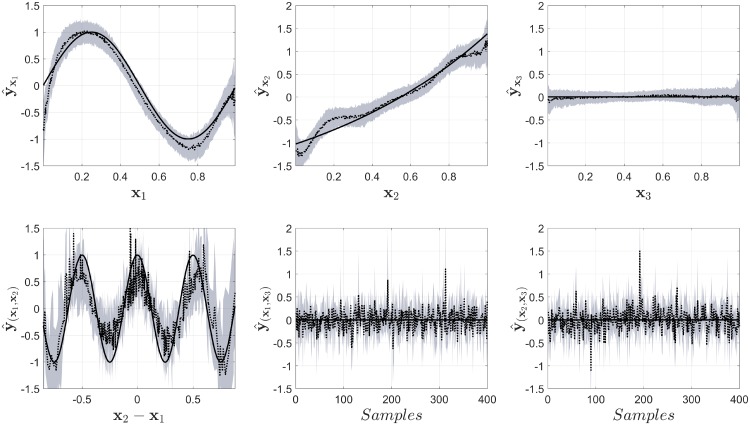
Output of the model for the decomposition using NObSP in the LS-SVM regression model with non-correlated inputs. The black solid line represents the true component, the dotted black line represents the mean for the estimations using NObSP, and the gray area represents the 95% confidence interval obtaining using bootstrap.

In order to evaluate whether the problem with the estimation was caused by the correlation between the input variables, we repeated the simulation using an input with uncorrelated inputs. The results are shown in Fig 6. It can be seen that COSSO-II now is able to retrieve, approximately, the functional forms for the variables *x*_1_ and *x*_2_, it also identifies that *x*_3_, as well as some interaction effects, do not have a contribution in the model. But, it still fails to identify the second-order interaction that was present in the original model. The output for the LS-SVM model using NObSP for this case is shown in Fig 7. The Fig shows that in this case NObSP can still retrieve the approximate functional forms, as shown previously.

**Fig 8:**
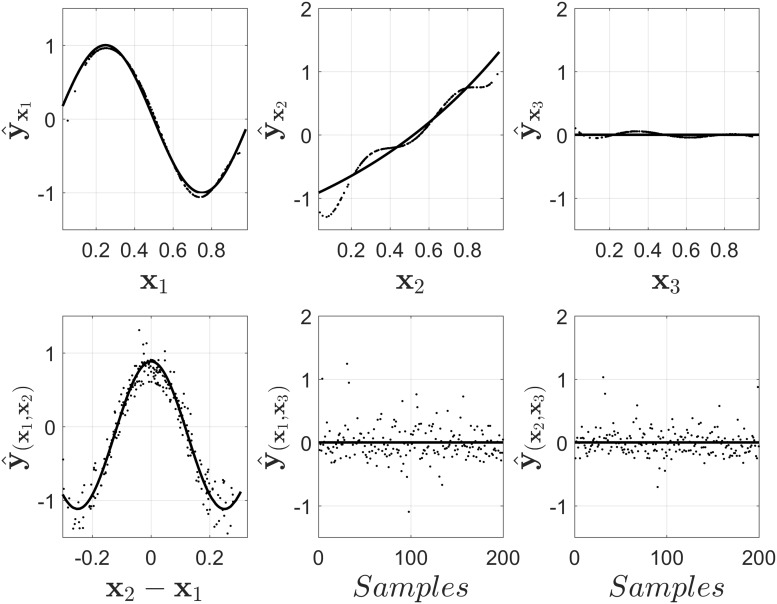
Results provided by NObSP using unseen data. The size of the test set was 250 samples. the samples were obtained in the same way as the training samples were produced. The solid line represents the reference functional forms, while the dots represent the output of the decomposition scheme.

**Fig 9:**
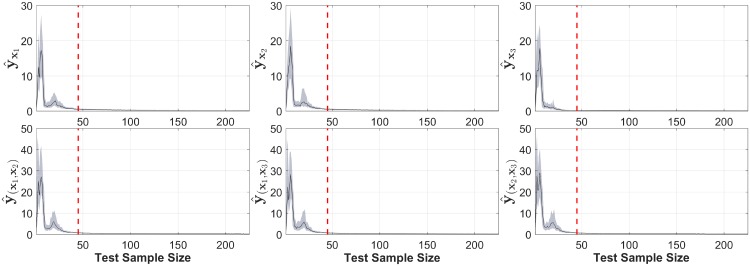
Convergence of the error between the real functional form and the estimated output provided by NObSP for different test set sizes. The solid line represents the median value and the shade area represents the 25 and 75 percentiles of the error. For each test set size 100 random simulations were performed. The red dashed line indicates the maximum rank obtained for the kernel matrices during training, which in this particular case was 45.

Additionally, the output for NObSP using the unseen test set can be seen in Fig 8 using 250 data points. The Fig shows that NObSP is able to retrieve the functional forms of the relationship between the input and the output of the model using new data samples. Furthermore, in Fig 9 we can see that the errors for the estimation of the functional components decrease as the size of the test set increases. This error converges around 45 samples, which is the maximum rank that was needed in order to construct appropriate projection matrices to decompose the output of the model.

### 3.3 Simulation study II: Concrete compressive strength dataset

In this section we used data from the concrete compressive strength database from the UCI machine learning repository. We tested the performance of NObSP and compared with COSSO-II. In this dataset there are 8 different input variables that are used in order to predict the strength of the concrete samples. The input variables are: the amount of cement, blast furnace slag, fly ash, water, superplasticizer, coarse aggregate, and fine aggregate, and the age of the cement in days. In total, the dataset contains 1030 samples. More information about the characteristics of the dataset can be found in [25]. For the simulations, we computed 100 realizations of the regression models, using random sampling with replacement. We express the output in terms of the mean and 95% confidence intervals.

**Fig 10:**
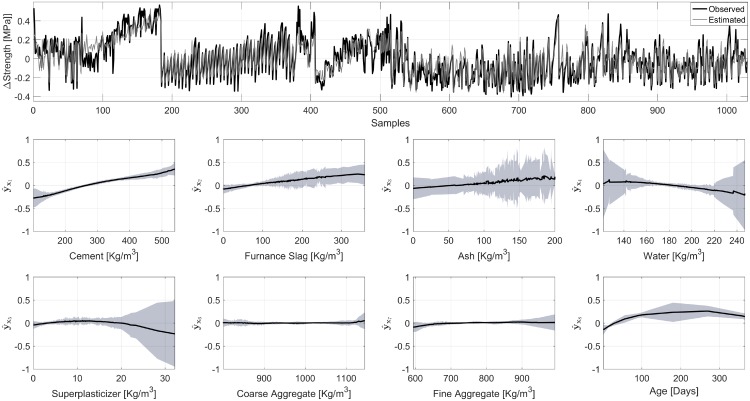
Model fitting, presented in the top plot, and estimation of the functional form of the contribution of the input variables in the concrete strength dataset from the UCI machine learning repository. In the top plot, the black line represents the measured strength of the cement mix, and in gray the estimated value using an LS-SVM model. For the contributions, the gray area represents the 95% confidence interval, obtained using bootstrap, and the solid black line the mean value of the contribution.

Fig 10 shows the results from NObSP. The Fig displays not only the decomposition but also the general performance for the regression model. Based on the magnitude of the responses, the elements that contribute the most to the strength of the concrete samples are: amount of cement, furnace slag, ash, and water. Other elements, such as superplasticizer, course and fine aggregate, do not contribute strongly to the final strength of the concrete sample.

## 4 Discussion

We have proposed an algorithm to decompose the output of an LS-SVM regression model into the sum of the partial nonlinear contributions, as well as interaction effects, of the input regressors. We have demonstrated that the functional form of the relation between the inputs and the output can be retrieved using oblique subspace projections. We have also shown that through appropriate kernel evaluations it is possible to find a proper basis for the subspace representing the nonlinear transformation of the input regression variables.

In contrast with other methodologies, the proposed algorithm does not require to define a-priori the model contributions for the decomposition. NObSP starts with a normal LS-SVM regression model, which solution, in terms of the kernel parameters, is used in order to implement the decomposition strategy. Other methodologies, such as COSSO, require to define a-priori whether the user is interested to decompose the output in terms of the main effects, or also to include second-order interactions. This might lead to different functional forms since there is no guarantee that both approaches will converge to the same solution. This was demonstrated in section 3.1 where COSSO-I and COSSO-II produced different outputs. In NObSP this is not a problem since the decomposition scheme does not require to solve an additional optimization problem.

The basis for the subspace spanned by the nonlinear transformation of the data, which is computed using kernel evaluations, can be seen as an *N*–dimensional approximation of the true subspace, which might be embedded in a much larger space. When using a linear kernel, it can be seen that the subspaces of the nonlinear transformations, as expected, cannot be properly constructed, resulting just in linear approximations of the true nonlinear model, as shown in Fig 3. When using a polynomial kernel, the decomposition scheme performs better than the linear case, being able to produce a better estimation of the nonlinear influences of the input regressors. However, as shown in section 3.1, the best results were obtained using the RBF kernel. This might be explained by the fact that the RBF kernel can be considered as an universal kernel, which is able to approximate the responses produced by any other kernel by changing its bandwidth.

In the second toy example, it was shown that NObSP was able to approximately identify the functional forms for the main effects, and second-order interaction effects on the output, in both cases when the input variables were correlated and uncorrelated. COSSO-II was only able to retrieve the main effects when the variables were not correlated. However, COSSO-II was able to identify more accurately the components that are not part of the original model.

When considering the concrete strength example, it can be seen that the decomposition scheme using NObSP produces results with small confidence intervals in most of the components. More importantly, the results provided seem to agree with the literature, where the strength of the concrete sample is expected to increase with increasing amounts of cement and decreasing amount of water, and it increases during the first days [25, 26]. Conversely, NObSP indicates that by increasing the amount of furnace slag, the strength of the cement increases, while Yeh et al., have reported the opposite [26]. However, the datasets used in both analyses are different, which might lead to different decompositions. In addition, it is important to notice that their analysis is done considering that several variables are kept constant, while in our case this restriction was not applied.

One of the main drawbacks of NObSP, in contrast with COSSO, is that it does not provide a closed form for the estimation of the nonlinear contributions and the interaction effects. However we have shown that it is possible to evaluate the decomposition scheme when a sufficient number of test samples are available. This is due to the fact that the decomposition is based on the projection of the observed data onto the respective subspace. Therefore, it is important that an adequate number of basis vectors is used in order to construct proper projection matrices. We have shown that the number of data points that are required in order to construct this projector is equal to the maximum rank of the kernel matrices that are created during training. In case that a closed form is required, one solution can be to introduce a multiple kernel learning problem similarly to the one proposed in [27]. This is out of the scope of this paper and will be addressed in future studies.

Additionally, there are some numerical issues that should be considered. Since kernel evaluations are needed to create a basis for the subspace spanned by the nonlinear transformation of a given variable, when using too many input regressors, it is possible that the computation of the Euclidean distances produce values close to machine precision. This is caused by the fact that in such cases several variables are set to 0, leaving only the variable of interest intact. In such cases, the results provided by NObSP are not reliable. Another problem that may arise is when the partial contribution of one variable on the output is much samaller than the contributions from other input variables or interaction effects. This will cause that this nonlinear contribution will not be found during the decomposition, since it will be absorved by the other components. In order to mitigate the impact of these issues we recommend to perform an initial input selection. In this way NObSP will be applied in a reduced set of input variables, this will not only select variables that might have a larger impact on the output, but will also reduce the risk of having numerical issues. Additionally, we also recommnd to normalize the input regressors to guarantee that the estimation of the distances is not biased by the magnitude of only one input variable.

We also performed simulations including a third order interaction component. The results from these simulation show that eventhoug NObSP was able to still retrieve the main effects and second order interaction effects, it failed to retrieve the third order interaction component. It produced results that were too noisy. This might be caused by the accumulation of errors when decomposing the target signal. It was not possible to compare the results from this simulation with other methods, since COSSO can only retrieve up to second oder interaction effects.

In summary, the advantages and disadvantages of NObSP and COSSO can be summarized as follows:

- NObSP does not require the define a priori the kind of relations of interest between the input regressors and the output. COSSO does require to define if the user is interested in the main effects or also in the interaction effects.
- NObSP converge to the solution whether the user is interested in finding the main and interaction effects. COSSO produces different results.
- NObSP is able to retrieve the functional forms even in the presence of correlated input regressors.
- COSSO produce models that are sparse. NObSP does not include sparsity in its definition.
- COSSO is able to retrieve close forms for the evaluation of new samples. NObSP, requires to construct projection matrices which require the evaluation of a minimum amount of points to produce a proper output.
- In contrast with COSSO, NObSP suffers of numerical issues due to the evaluation of kernel functions with vectors that contain a considerable amount of zeros.

NObSP can also be extended to the analysis of dynamical systems. For instance, NObSP can be introduced naturally for the nonlinear identification of dynamical systems using subspace identification techniques such as the one proposed in [28]. In the actual formulation, the relation between the input variables and the output in the regression model is considered static. However, by using as input variables a Hankel expansion, or any other expansion, of the input regressors, it is possible to consider a dynamic nonlinear model, introducing this framework in the field of system identification.

Finally, it is interesting to notice that NObSP relies only on the tuning of a kernel in order to decompose the output in the partial nonlinear contributions of the input and the interaction effects. This provides insights about the geometry of the regression models using kernels, by linking the space where the original data is embedded and the subspaces generated by the nonlinear transformation of the data. This also indicates that this methodology is not limited to the use in LS-SVM model, but it can be adapted to any kind of kernel-based regression models, where the output can be represented as a linear combination of kernel evaluations, like in equation Eq (4). Such models include kernel principal component regression [29, 30], kernel partial least squares [31], and kernel ridge regression [32].

## 5 Conclusions

In this manuscript we proposed a decomposition scheme using oblique subspace projections, called NObSP, which is able to retrieve relevant information about the input variables and their relationship with the output in an LS-SVM regression model, facilitating its interpretation. The performance of the proposed model was demonstrated using 2 different toy examples as well as a practical example from a public database. This methodology has a huge potential in many fields, including biomedical applications. For instance, it can be used for the study of the interactions between different physiological signals, providing extra information for the understanding of some underlying regulatory mechanisms, and supporting clinical diagnosis and treatment.
